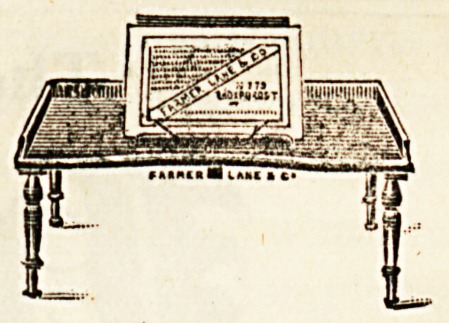# Practical Departments

**Published:** 1900-04-07

**Authors:** 


### PRACTICAL DEPARTMENTS.

### INVALID FURNITURE.

(MESSRS. FARMER, LANE, AND CO.)

THE demand for furniture and appliances for the use of the sick is probably fairly equally maintained throughout the year, but it is often when the first bright weather makes its appearance that the overhauling of old "stock" takes place, and chairs and comforts for those convalescents able to sit in balconies and gardens are once more in request. Messrs. Farmer, Lane, and Co., 77 and 79, New Oxford Street, are showing this spring some very excellent lounge chairs suitable for the above purpose; made in cane they are very light, roomy, and comfortable, and the price is moderate enough to recommend them to all. That of the chair here illustrated runs from 23s. 6d. to 2 guineas. The position can be altered, the back is adjustable to any single angle, and the leg rest extends if desired, being tucked away beneath the seat when not required. In consequence of the war, which like King Charles's head, simply cannot be kept out of anything one writes these days, there is naturally a great demand for invalid comforts from all over the country for the benefit of the sick and wounded heroes being nursed back to health here in England. Messrs. Farmer, Lane, and Co. have just completed a large order for one of the military hospitals for back rests such as the one illustrated. We have recommended this rest before as being admirably adapted for its special purpose, and are certain that it will give satisfaction wherever tried. Another sick-room necessary is the indispensable bed table, of which perhaps the most useful variety is that made with a folding book ledge, a real boon to invalids, enabling them to read with a minimum of fatigue. The legs unscrew, so that for packing purposes the little table can be reduced to a flat board. Messrs. Farmer, Lane, and Co. have also many kinds of carrying and propelling chairs, of all designs and prices; bed frames or "cradles" made in cane; lounge and wheel chairs and couches of every make and kind. Inspection of these should certainly be made by anyone in search of invalid furniture for hospital ward or private sick-room.

## Figures and Tables

**Figure f1:**
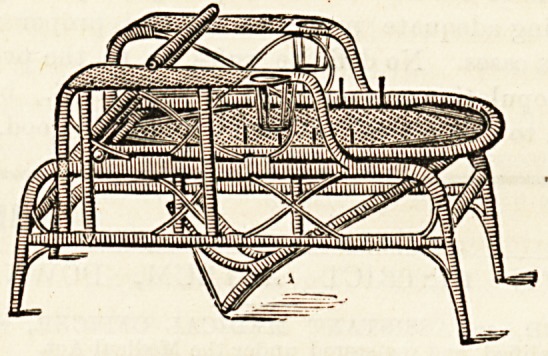


**Figure f2:**
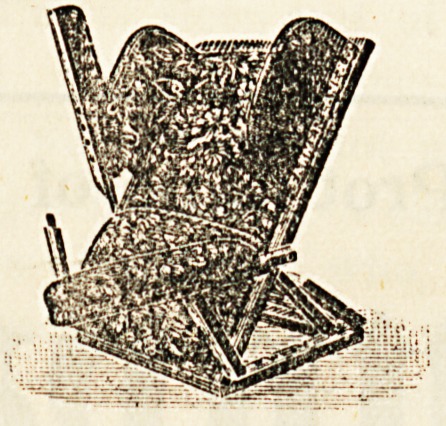


**Figure f3:**